# An initial comparative map of copy number variations in the goat (*Capra hircus*) genome

**DOI:** 10.1186/1471-2164-11-639

**Published:** 2010-11-17

**Authors:** Luca Fontanesi, Pier Luigi Martelli, Francesca Beretti, Valentina Riggio, Stefania Dall'Olio, Michela Colombo, Rita Casadio, Vincenzo Russo, Baldassare Portolano

**Affiliations:** 1 DIPROVAL, Sezione di Allevamenti Zootecnici, University of Bologna, Via F.lli Rosselli 107, 42123 Reggio Emilia, Italy; 2 Biocomputing Group, Computational Biology Network, Dept. of Biology, University of Bologna, Via San Giacomo 9/2, 40126 Bologna, Italy; 3 Dept. S.En.Fi.Mi.Zo., Sezione di Produzioni Animali, University of Palermo, Viale delle Scienze - Parco d'Orleans, 90128 Palermo, Italy

## Abstract

**Background:**

The goat (*Capra hircus*) represents one of the most important farm animal species. It is reared in all continents with an estimated world population of about 800 million of animals. Despite its importance, studies on the goat genome are still in their infancy compared to those in other farm animal species. Comparative mapping between cattle and goat showed only a few rearrangements in agreement with the similarity of chromosome banding. We carried out a cross species cattle-goat array comparative genome hybridization (aCGH) experiment in order to identify copy number variations (CNVs) in the goat genome analysing animals of different breeds (Saanen, Camosciata delle Alpi, Girgentana, and  Murciano-Granadina) using a tiling oligonucleotide array with ~385,000 probes designed on the bovine genome.

**Results:**

We identified a total of 161 CNVs (an average of 17.9 CNVs per goat), with the largest number in the Saanen breed and the lowest in the Camosciata delle Alpi goat. By aggregating overlapping CNVs identified in different animals we determined CNV regions (CNVRs): on the whole, we identified 127 CNVRs covering about 11.47 Mb of the virtual goat genome referred to the bovine genome (0.435% of the latter genome). These 127 CNVRs included 86 loss and 41 gain and ranged from about 24 kb to about 1.07 Mb with a mean and median equal to 90,292 bp and 49,530 bp, respectively. To evaluate whether the identified goat CNVRs overlap with those reported in the cattle genome, we compared our results with those obtained in four independent cattle experiments. Overlapping between goat and cattle CNVRs was highly significant (P < 0.0001) suggesting that several chromosome regions might contain recurrent interspecies CNVRs. Genes with environmental functions were over-represented in goat CNVRs as reported in other mammals.

**Conclusions:**

We describe a first map of goat CNVRs. This provides information on a comparative basis with the cattle genome by identifying putative recurrent interspecies CNVs between these two ruminant species. Several goat CNVs affect genes with important biological functions. Further studies are needed to evaluate the functional relevance of these CNVs and their effects on behavior, production, and disease resistance traits in goats.

## Background

The goat (*Capra hircus*) represents one of the most important farm animal species. It is reared in all continents with an estimated world population of about 800 million of animals and about 560 breeds, which constitute approximately 12% of the total number of recorded domesticated mammalian livestock breeds of the world [1]. The diffusion of this species is mainly due to its capacity to supply milk, meat, and fibers for human consumption and use in a large number of different environments, including those poor of vegetation. In general, goat breeding represents an essential support for marginal economies in most developed and developing countries.

Despite the importance of this species, studies on the goat genome are still in their infancy compared to those in other farm animal species. A first and a second generation genetic maps of the goat genome have been obtained by Vaiman et al. [2] and Schibler et al. [3] by mapping a few hundred microsatellite markers in half-sib paternal goat families, with about 90% genome coverage. The relatively short evolutionary time separating the goat from the cattle and sheep [4-6] made it possible to use microsatellites developed in these two species to successfully genotype goats, even though interspecific priming often resulted in a marked loss of heterozygosity [2,3]. A comparative cytogenetic map of the goat genome has been developed using cattle and sheep BAC clones [3]. This map has been improved adding many other physically mapped genes, as recently reviewed in a compiled list including 268 genes and 144 microsatellites, roughly including 65% of the goat chromosome bands [7]. Comparative mapping between cattle and goat (both species have 2n = 60) has shown only few rearrangements in agreement with the similarity of chromosome banding [7,8].

Analysis of the goat genome provided a few important findings including the positional cloning of the polled intersex syndrome (PIS) locus located on goat chromosome (CHI) 1q43 and determined by a deletion of 11.7 kb containing mainly repetitive sequences [9,10]. Additional studies have been focused on milk protein gene polymorphisms and their effects on milk production traits (e.g. [11-13]). Polymorphisms in the goat *PRNP *gene have been associated with susceptibility to scrapie in different breeds [14]. Few other studies reported QTL for milk and fleece production traits and disease resistance [15-20]. Investigations of genes affecting coat colour identified polymorphisms associated with this phenotypic trait [21,22]. In particular, the *Agouti *locus in goat was shown to be highly variable including missense mutations and copy number variation (CNV) [22].

Recent studies have shown that copy number variants, defined as intraspecific gains or losses of ≥ 1 kb of genomic DNA [23,24], represent an important source of variability of mammalian genomes (~0.4-25% of the genome) as reported in human (e.g. [25-34]), chimpanzee [35,36], rhesus macaque [37], mouse [38-42], rat [43,44], dog [45,46], pig [47], and cattle [48-52]. CNVs can change gene structure and dosage, can regulate gene expression and function and for these reasons they have potentially more effects than the most frequent single nucleotide polymorphisms (SNPs) in determining phenotypic differences [43,53-56]. CNVs can represent benign polymorphic variants even if in many other cases they are associated with human Mendelian and complex genetic disorders (reviewed in [57,58]). In farm animals, several traits are caused by CNV affecting genes or gene regions. For example, the *Dominant white *locus in pigs includes alleles determined by duplications of the *KIT *gene [59,60]. CNV also affects the *Agouti *locus in sheep and goats and contributes to the variability of coat colour in these two species [22,61]. CNV in intron 1 of the *SOX5 *gene causes the pea-comb phenotype in chicken [62] and the late feathering locus in this avian species includes a partial duplication of the *PRLR *and *SPEF2 *genes [63].

Genome-wide discovery and frequency evaluation of CNVs have been possible with the development of high-resolution array comparative genome hybridisation (aCGH) and, subsequently, with data analysis of high-density SNP platforms and paired end and deep sequencing approaches [64-69]. An advantage of aCGH is that hybridisation can be performed using heterologous DNA, i.e. genomic DNA of a different species but close to that used to develop the array, taking advantages from completely sequenced, assembled, and richly annotated genomes. Cross species aCGH experiments have been successfully applied using human arrays to analyse CNVs in chimpanzee and other primates [35,36,70], and using chicken based arrays to identify CNVs in turkey [71], duck [72], and zebra-finch [73] genomes.

Here we designed a cross species cattle-goat aCGH experiment in order to identify CNVs in goats of different breeds (both cosmopolitan and local) using information of the cattle genome and we obtained a first comparative map of CNVs of the *Capra hircus *genome.

## Results and discussion

### Identification of goat CNVs and comparative analysis between goat and cattle CNVRs

**Figure 1:**
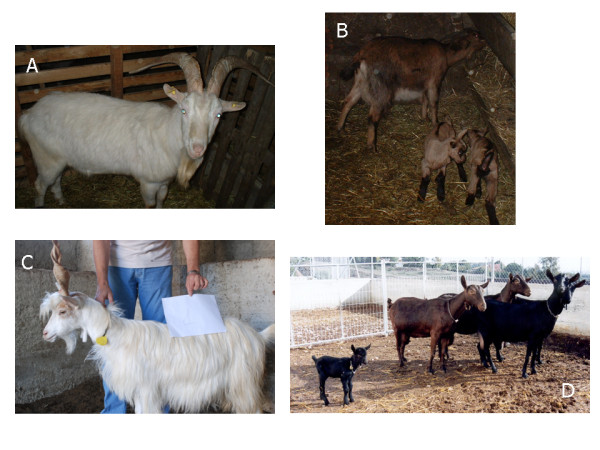
**Goat of different breeds used for CNVs discovery**. A = Saanen; B = Camosciata delle Alpi; C = Girgentana; D = Murciano-Granadina.

The goat genome has not been sequenced yet and the cattle is the closest species to the goat for which an assembled genome is available [74,75]. In order to give a preliminary evaluation of the extent of sequence identity between these two ruminant species, we compared goat genomic sequences longer than 1 kb available in EMBL database with homologous cattle genomic regions. The average sequence identity between these two species was 82.79% (over 166.8 kb of aligned sequences), that increased at 93.77% if only exonic sequences (32.5 kb) were considered (data not shown). Even if this rough evaluation cannot give a complete picture of sequence divergence between cattle and goat, it indicates that cattle-goat cross-species DNA hybridization is possible as also demonstrated in physical mapping of cattle BAC and YAC clones to the goat genome and *vice versa *as well as by interspecific use of microsatellites [2,3,7,76]. Therefore, to identify CNVs in goats, we carried out a cross-species aCGH experiment using a cattle (*Bos taurus*) custom tiling array including ~385,000 oligonucleotide probes and goat genomic DNA obtained from 9 goats of four different breeds (3 Saanen, 1 Camosciata delle Alpi, 3 Girgentana, and 2 Murciano-Granadina goats), chosen according to their differences in production and morphological traits, and origin (Figure 1). Saanen is the most popular dairy breed that was originated in Switzerland, highly selected for heavy milk production and with completely white/cream coat colour. Camosciata delle Alpi is an Alp mountain breed of the Chamois group. Girgentana is a Sicilian breed in an endangered status that is probably of Afghan and Himalayan origin, well adapted to the dry Sicilian environment and with very mild behavior. Girgentana goats have long corkscrew horns and cream/light-grey coat colour with, usually, a few small red spots around eyes and ears. Murciano-Granadina is a native Spanish breed with two colour types, solid black or solid brown (*caoba*).

Specific criteria were used to call CNVs in this cross-species aCGH experiment. CNVs were reported using 10 different algorithms developed for data segmentation and averaging their results as implemented in the CGHweb server [77]. The averaged log_2 _ratio threshold used to call CNVs was empirically determined considering the number of false positives called in the reference DNA self-self hybridization and the validation obtained by semiquantitative fluorescent multiplex PCR (SQF-PCR) (see Methods and data reported below for details). Applying these criteria, on the whole we identified 161 CNVs (Table 1 and Additional file 1). The largest number of CNVs was identified in the Saanen breed and the lowest number was reported for the Camosciata delle Alpi analysed goat. About 74% of the identified CNVs were observed in only one breed. On average each sample contained 17.9 CNVs. This result is comparable to what has been obtained in similar aCGH experiments that analysed the cattle [51], dog [45], and chicken [78] genomes, in which 11.6, 17.2 and 9.6 CNVs were evidenced for each animal in the three species, respectively (Table 2). Two CNVs (not overlapped to any other CNV) were also called in the self-self hybridization providing a rough estimation of the false discovery rate (FDR) among the set of identified CNVs (FDR = 11%). However, technical issues, like sequence divergence between the reference genome and the hybridized DNA and heterogeneity of DNA quality among different samples, make it difficult to precisely estimate the experimental FDR. The estimated FDR using the self-self hybridization data in this cross-species experiment is a little bit larger than that obtained with homologous DNA hybridization in aCGH. Indeed, in an aCGH study in dog, FDR was about 3% due to only one CNV detected in the self-self hybridization [46]. However, in another experiment carried out in humans, FDR was estimated to be 8-24% [28].

**Table 1:** Summary of CNVs identified in the analysed goat breeds

Breed (no. of animals)	Number of CNVs				CNV average size (kb)
		
	Total	Unique	Gain	Loss	
Saanen (3)	62	50	24	38	73.1
Camosciata delle Alpi (1)	8	5	4	4	122.4
Girgentana (3)	54	37	20	34	118.4
Murciano-Granadina (2)	37	27	11	26	107.3
Total (9)	161	119	59	102	98.6

**Table 2:** Comparison between this and other similar CNV studies using aCGH in mammalian and avian species.

Species	No. of individuals	aCGH platforms	Mean probe spacing (kb)	Total no. of CNVs	Mean no. of CNVs per individual	Total no. of CNVRs	CNVR mean size (kb)	References
Goat	9	385 k oligo aCGH	6.3	161	17.9	127	90.3	This study
Cattle	90	385 k oligo aCGH	~6	1,041	11.6	177	158.6	[51]
Cattle	20	6.3 million oligo aCGH	0.4	-	-	304	72.0	[52]
Human	270	BAC aCGH^2^	-	-	-	913	228.0	[28]
Human	40	42 million oligo aCGH	0.06	51,997	1300	-	2.9^6^	[34]
Chimpanzee	20	BAC aCGH^2^	-	355	17.8	-	-	[35]
Macaque	9	385 k oligo aCGH	6.5	214	21.4	123	101.2	[37]
Dog	9	385 k oligo aCGH	4.7^3^	155	17.2	60	309.5	[45]
Mouse	21^1^	385 k oligo aCGH	~5	80	2-38^5^	-	271.5	[39]
Mouse	20^1^	2.1 million oligo aCGH	1	10,681	26.4-48.3^5^	3,359	64.0	[54]
Rat	3	385 k oligo aCGH	~5^4^	33	11	33	256.0	[43]
Chicken	10	385 k oligo aCGH	2.6	96	9.6	-	166.7	[78]

^1 ^No. of strains (2-6 individuals per strain).

^2 ^Whole Genome TilePath array comprising 26,574 large insert clones.

^3 ^Median probe spacing.

^4 ^Considering nonrepetitive parts of the genome.

^5 ^Depending on the strain.

^6 ^Median size.

**Figure 2:**
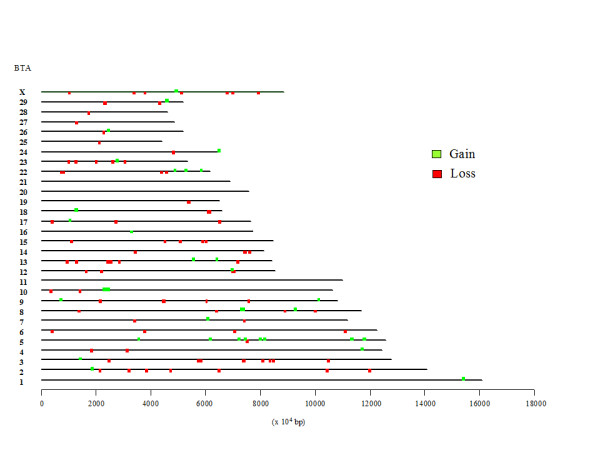
**Comparative map of CNVRs identified in goats reported on the bovine chromosomes**.

CNV regions (CNVRs) were determined by aggregating overlapping CNVs identified in different animals as previously reported [28,51] and considering a conservative approach due to the specificity of our experiment (see Methods for details). On the whole, we detected 126 CNVRs covering about 11.39 Mb of the virtual goat genome referred to the bovine genome, version Btau_4.0 (Figure 2 and Additional file 2). This fraction corresponds to 0.432% of the latter genome, considered adding bases in the 29 autosomes and the X chromosome assembled in the Btau_4.0 version (11.39 Mb/2634 Mb). The chrUnAll (unassembled scaffolds) of the Btau_4.0 version was not included in the tiling array due to difficulties in interpreting the results that might have been derived by the short assembled fragments and mapping uncertainty [51]. In addition, the tiling arrays included 4,673 oligonucleotides designed on a portion of BTA13 (from nucleotide position 48 Mb to nucleotide position 78 Mb) derived from the UMD *Bos taurus *v. 2.0 assembly [75]. This additional BTA13 portion was included in the tiling arrays because the agouti signaling protein (*ASIP*) gene was not correctly assembled in the Btau_4.0 version of BTA13 and was reported in unassembled scaffolds only. This UMD 2.0 BTA13 region was added as an internal control because we previously demonstrated that the *ASIP *gene and the close S-adenosylhomocysteine hydrolase (*AHCY*) gene are included in a goat CNVR [22]. This CNVR may represent a recurrent interspecies CNVR since the same two genes are involved in a large duplicated region in sheep [61]. Considering this additional CNVR, on the whole we identified 127 CNVRs for a total of 11.47 Mb (Figure 2 and Additional file 2). These 127 CNVRs included 86 losses and 41 gains, whereas none reported both events. Of these CNVRs, 14 were found in multiple animals of different breeds (no. = 7) or in multiple animals of the same and different breeds (no. = 6) or in multiple animals of the same breed only (no. = 1). All other events (no. = 113) were found only in one animal. The regions that were affected by these gain or loss events in goats ranged from 24,605 bp (BTA22, CNVR no. 101) to 1,075,778 bp (BTA17, CNVR no. 90) with mean and median equal to 90,292 bp and 49,530 bp, respectively (Additional file 2). Using a similar aCGH experiment Liu et al. [51] reported that cattle CNVRs are on average a little bit larger than those we reported in goats (mean and median equal to 159,031 bp and 89,053 bp, respectively). This is in contrast to Fadista et al. [52] who, using more dense arrays in another aCGH experiment reported a median size of CNVRs equal to 16.7 kb. Mean and median differences between goat and cattle experiments might be due to i) the cross species experiment performed in goat that might not be able to correctly identify the borders of the CNVRs, ii) the oligonucleotide density in the aCGH experiments, iii) true differences between species/breeds. It is interesting to note that the number of loss events was about two fold the number of gain events in both cattle and goat. This finding might be derived by both biological and technical reasons. Non-allelic homologous recombination, which seems to be one of the most important mechanisms responsible for CNV formation, has been shown to generate more deletions than duplications [79]. On the other hand, aCGH detection methods seem to favor the identification of deletions as reported by several other studies [28,47,51,52].

Additional file 3 reports the extension of CNVRs distributed for the different bovine chromosomes used in the comparative analysis with the goat genome. In only three chromosomes (BTA11, BTA20, and BTA21) we did not identify any CNVRs. BTA5 included the largest number of CNVRs (no. = 11), whereas BTA17, BTA10, and BTA18 included the largest extension of regions affected by CNVs (1.6%, 1.3% and 1.0% of their length, respectively) (Figure 2). In cattle a similar aCGH experiment (that however included a larger number of animals [51]) showed the greatest enrichment for CNVRs on BTA5, BTA15, BTA18, BTA27, BTA29, and BTAX. Comparative mapping and chromosome banding similarities between cattle and goat indicate highly conserved synteny between these two ruminant species even if a few rearrangements have been evidenced mainly on CHI14 containing a small BTA9q11-q13 segment, and some other gene order rearrangements for chromosomes CHI2 compared to BTA2, CHI19 compared to BTA19, and CHIX compared to BTAX [7,8]. CNVRs were evidenced in these interested chromosome regions. However, it is not possible to evidence if CNVs are precisely positioned in rearranged regions because of the low resolution of the rearrangements so far described between goat and cattle chromosomes due to the few mapped genes.

To evaluate if CNVRs we identified in goats overlap with CNVRs reported in cattle, we compared our results with those obtained in four independent cattle experiments [49-52] carried out i) using aCGH including ~385,000 tiling oligonucleotides (177 CNVRs [51]) or ii) including 6.3 million of probes (304 CNVRs [52]) and iii) using the Illumina BovineSNP50 BeadChip containing about 50K SNPs as reported by Matukumalli et al. ([49]; 79 CNVRs) and by Bae et al. ([50]; 368 CNVRs) (Additional file 4 and Additional file 5). Overlapping between aCGH results obtained in goat and cattle was highly significant (P < 0.0001) for both cattle experiments (17 and 11 goat CNVRs overlapped with cattle CNVRs identified by Liu et al. [51] and Fadista et al. [52], respectively). Only two goat CNVRs matched cattle CNVRs identified with the SNP panel [49,50], therefore overlapping was not significant. A similar bias on the common CNVRs between aCGH and SNP genotyping experiments was also evident comparing the cattle data obtained by the two methods (Additional file 5 and [51]). This could be due to resolution and genome coverage differences between the two platforms [51]. However, merging all cattle CNVRs reported in the four different experiments (on the whole 764 unique cattle CNVRs were obtained [49-52], Additional file 4), overlap with goat CNVRs was highly significant (P < 0.0001), confirming the results obtained considering the different cattle datasets separately (Additional file 5).

As the goat genome is not sequenced yet, we could not evaluate if the goat CNVRs have similar sequence characteristics in goat and cattle. Segmental duplications have been shown to significantly overlap with CNVRs in cattle [51,52] as well as in several other species [31,32,34,35,37,39,46]. As segmental duplications might facilitate non-allelic homologous recombination, it is likely that they are as well involved in the mechanisms that produce CNVs in goats. The overlapping CNVRs between goat and cattle might represent homoplastic recurrent interspecies CNVRs probably driven by genomic regions prone to instability present in the cattle-goat common ancestor that might have been retained in the genomes of both extant species. Indeed, cattle and goat share a common ancestor in the early Miocene about 17-23 Million years ago [4-6]. Similar reasoning could be considered for the CNVR that includes the *ASIP *gene [22] for which a recurrent CNVR has been reported in sheep [61], but not in cattle. Sheep and goat lineages separated about 6-14 millions of years before present [80]. Significant overlap of CNVRs among different species has been also observed comparing the human with both chimpanzee and rhesus macaque genomes [35-37]. These two non-human primate species diverged from the human lineage about 6 and 25 million years ago [81,82]. Together these results suggest that certain genomic regions are prone to recurrent CNV formation and instability in both the primate and the *Artiodactyla *evolutionary lineages. However, a change in the formation process of CNVs and segmental duplications in the human genome might be occurred in recent evolutionary history [83]. It will be interesting to evaluate if this has occurred in other lineages. A comparative analysis of CNVRs identified in cattle, goat, and sheep can open perspectives to evaluate the evolutionary mechanisms determining CNV formation during the mammalian evolution.

### Validation and gene content of CNVRs

Several results suggest most of our CNVs are correctly identified. First of all, the number of CNVs was lower in the analysed Camosciata delle Alpi goat (no. = 8; Table 1). This was expected because the aCGH reference was a sample of genomic DNA of another goat of the same breed. Similar results were also reported in mouse and dog aCGH studies that used a reference genomic DNA of an animal of the same breed/line of others that have been analysed for CNV discovery [39,45]. In addition, an internal control included in the design of the tiling arrays was represented by a portion of BTA13 derived from the UMD 2.0 assembly that contains the *ASIP *and *AHCY *genes that we previously showed to be affected by CNV in goats [22]. All goats that were shown to have multiple copies of the *ASIP *and *AHCY *genes with different methods [22] reported evidence of gain in the aCGH experiment, compared to the reference Camosciata delle Alpi genomic DNA. The results for this region were also used to set an empirical threshold to call CNVs by using the CGHweb platform and multiple algorithms [77] (see Methods).

**Figure 3:**
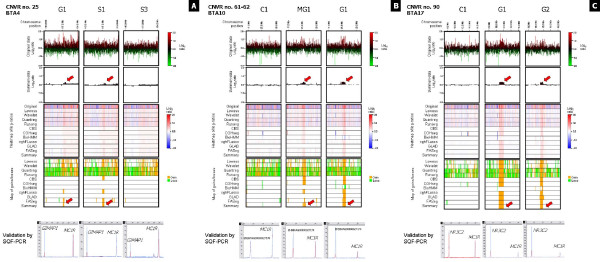
**aCGH and SQF-PCR results for CNVRs nos. 25 (A), 61-62 (B), and 90 (C)**. For each CNVR, results were reported for three goats indicated at the top in the correspondence of the related charts and images (C1 = Camosciata delle Alpi, animal no. 1; G1 = Girgentana, animal no. 1; G2 = Girgentana, animal no. 2; MG1 = Murciano-Granadina, animal no. 1; S1 = Saanen, animal no. 1; S3 = Saanen, animal no. 3). For the aCGH experiment, images have been reported for i) log_2 _ratio plot of original data, ii) log_2 _ratio plot of summary data (pointwise averaging of all computed profiles), iii) heatmap of log_2 _ratios for original, smoothed/segmented, and summary data, and iv) maps of gains/losses for smoothed/segmented and summary data (gain is indicated in orange, loss is indicated in green). Red arrows indicate regions of copy gain. Smoothed/segmented data were obtained with several algorithms (Lowess, Wavelet, Quantreg, ruavg, CBS, CGHseg, BioHMM, cghFLasso, GLAD, and FASeg) averaged in the summary data [77].. Semiquantitative fluorescent multiplex PCR (SQF-PCR) electropherograms for fragments of three CNVRs are superimposed on that of the reference Camosciata delle Alpi goat after normalization against the control *MC1R *amplicon. Results obtained using *DGAT1 *for normalization are overlapping with those obtained with *MC1R *and for this reason are not reported.

Other three CNVRs identified on the goat chromosomes corresponding to BTA4, BTA10, and BTA17 (Table 3 and Additional file 6) were directly validated with SQF-PCR as reported for the *ASIP *and *AHCY *genes [22]. The CNVR of BTA4 (CNVR no. 25) included a gene coding for the GTPase, IMAP family member 1 (*GIMAP1*; ENSBTAG00000001198) that was used to design the primers for PCR validation. Semiquantitative fluorescent multiplex PCR confirmed the gain of copies already reported in the goats used in the aCGH experiment and in additional goats of the Saanen, Girgentana and Murciano-Granadina breeds (Figure 3A) (Additional file 7). The evaluated CNVR of BTA10 (CNVR no. 61) was originally identified in a Murciano-Granadina goat by aCGH (Additional file 1 and Additional file 2). The bovine sequence coding for an unknown transcript (ENSBTAG00000027170) positioned in this region was used to design PCR primers for validation. However, analyzing the bovine genome by BLAT using the ENSBTAG00000027170 sequence, a large number of significant hits (E-value < 4E-233, identity > 89%) were obtained, and the hits included only sequences assembled in the BTA10 encompassing the goat CNVRs no. 61 and 62 or included in unassigned scaffolds (data not shown). These regions of BTA10 have been already reported to contain CNVs in different cattle breeds [51,52] (Additional file 4). Analyzing by SQF-PCR goats other than those used for aCGH, we identified that this complex region (CNVRs no. 61 and 62) is affected by both gain and loss of DNA copies that did not occur specifically in the evaluated goat breeds (Additional file 7). Therefore, even if these CNVRs were classified as gain (Figure 3B) (Additional file 2), they should be considered together as gain/loss CNVRs. CVNR no. 90 on BTA17 (the largest we identified) includes the nuclear receptor subfamily 3, group C, member 2 (*NR3C2*) gene (ENSBTAG00000027182). PCR primers were tested on additional animals as reported for the previous two CNVRs. Only and all Girgentana goats showed gain of DNA copies in this region (Figure 3C), including the three Girgentana goats used for aCGH analyses (Additional file 7), even if the signal for one of them did not trespass the averaged log_2 _ratio threshold of 0.175. The *NR3C2 *gene encodes the mineralocorticoid receptor, which mediates aldosterone actions on salt and water balance within distal nephron cells, with crucial effects on blood pressure and potassium homeostasis. The protein acts as a ligand-dependent transcription factor that binds to mineralocorticoid response elements in order to transactivate target genes. Mutations in this gene in humans cause autosomal dominant pseudohypoaldosteronism type I, a disorder characterized by renal resistance to aldosterone as well as salt wasting, dehydration, hyperkalemia, metabolic acidosis and failure to thrive in the newborn [84]. Defects in this gene are also associated with early onset hypertension [85], whereas overexpression of this gene in forebrain decreases anxiety-like behavior [86]. It is tempting to speculate that additional copies of the *NR3C2 *gene (if functional) could contribute to specific adaptation traits to harsh and dry environments and to the very mild behavior of the Girgentana goats compared to other breeds.

**Table 3:** Validated goat CNVRs using semiquantitative fluorescent multiplex-PCR (SQF-PCR)

**CNVR no**.	Chromosome	BTA coordinates (Btau_4.0)	Target gene symbol (Ensembl entry no.)	Gain/Loss in aCGH analyses	Gain/Loss in SQF-PCR analyses
25	4	117225479-117366050	*GIMAP1 *(ENSBTAG00000001198)	Gain	Gain
61-62	10	23334056-23640961 23739149-24536154	(ENSBTAG00000027170)	Gain	Gain/Loss
76^1^	13	64082600-64157186^2^	*ASIP *(ENSBTAG00000034077)	Gain	Gain
76^1^	13	64082600-64157186^2^	*AHCY *(ENSBTAG00000018101)	Gain	Gain
90	17	10532314-11608092	*NR3C2 *(ENSBTAG00000027182)	Gain	Gain

^1 ^Reported in [22].

^2 ^Coordinates of the BTA13 of the UMD 2.0 *Bos taurus *genome assembly.

Considering all 127 goat CNVRs, 78 of them (61.4%) partially or completely spanned cattle Ensembl annotated genes (Btau_4.0 version), including 249 Ensembl peptides, corresponding to 199 unique Ensembl cattle genes, representing 261, 126, and 864 gene ontology (GO) categories for molecular function, cellular components and biological processes, and 870 different PANTHER terms (Additional file 8 and Additional file 9). For example, considering GO annotation for biological processes, several GO categories are significantly (*P *< 0.001) over-represented in goat CNVRs (Table 4; Additional file 10). A few of these GO terms (response to stimulus; defense response) have been also already reported to be over-represented in CNVRs of the mouse and human genomes [39,87]. In general, goat CNVRs resulted particularly enriched in "environmental" functions (Table 4; Additional file 10 and Additional file 11) as already reported in other species [39,46,50-52,87]. This indication might be important in understanding the evolutionary and selection processes that determined the occurrence and diffusion of this variability in the goat genome. It could be possible that positive selection on duplications (based on functional effects) has occurred for these particular gene categories or that these enrichments might instead have been arisen from nonuniform negative selection on gene copy changes. This could be due to the fact that duplication or deletion of nonenvironmental genes might be more frequently deleterious than copy number changes of environmental genes [88]. The complex domestication process and artificial selection for breeding purposes (including bottleneck and expansion) that largely contributed to establish goat breed differences [89] might further complicate the evaluation of these two different hypotheses.

**Table 4:** Gene ontology (GO) categories significantly overrepresented (FDR, *P *< 0.001) in goat CNVRs.

GO level	GO term	Go name	No. in goat CNVRs	**Expected no**.
***Molecular Function***	GO:0005525	GTP binding	24	3.7
	GO:0032561	guanyl ribonucleotide binding	24	3.8
	GO:0003924	GTPase activity	16	2.1
	GO:0019001	guanyl nucleotide binding	24	3.9
	GO:0017111	nucleoside-triphosphatase activity	25	8.4
	GO:0016462	pyrophosphatase activity	25	8.6
	GO:0016818	hydrolase activity, acting on acid anhydrides, in phosphorus-containing anhydrides	25	8.6
	GO:0016817	hydrolase activity, acting on acid anhydrides	25	8.8
	GO:0015171	amino acid transmembrane transporter activity	6	0.50
	GO:0004869	cysteine-type endopeptidase inhibitor activity	5	0.37
	GO:0017076	purine nucleotide binding	37	19
	GO:0046943	carboxylic acid transmembrane transporter activity	6	0.73
	GO:0005342	organic acid transmembrane transporter activity	6	0.74
	GO:0042974	retinoic acid receptor binding	3	0.094
	GO:0046965	retinoid X receptor binding	3	0.094
	GO:0022804	active transmembrane transporter activity	12	3.3
	GO:0032553	ribonucleotide binding	37	18
	GO:0032555	purine ribonucleotide binding	37	18
	GO:0005275	amine transmembrane transporter activity	6	0.55
Biological processes	GO:0050896	response to stimulus	41	15
	GO:0006952	defense response	16	2.6
	GO:0006950	response to stress	25	8.0
	GO:0051704	multi-organism process	13	2.2
	GO:0046942	carboxylic acid transport	8	0.76
	GO:0015849	organic acid transport	8	0.77
	GO:0051707	response to other organism	10	1.5
	GO:0009617	response to bacterium	9	1.1
	GO:0042742	defense response to bacterium	7	0.68
Cellular components	GO:0042613	MHC class II protein complex	5	0.14
	GO:0042611	MHC protein complex	7	0.62

The complete list of overrepresented GO and PANTHER categories is reported in Additional file 10 and Additional file 11, respectively, including details about the categories of the whole cattle genome (Btau_4.0 version) used as reference.

Considering the 199 unique Ensembl cattle genes included in the CNVRs identified in goats, for 119 we retrieved a human orthologous gene. Mutations in only 8 of these genes cause Mendelian disorders or are associated with genetic diseases in human (Additional file 12). None of these 199 cattle genes is involved in any reported genetic disease in goat, sheep or cattle. Among the goat genes already mapped [7] only one (*TTN*) is included in a CNVR (Additional file 12).

Few QTL studies have been reported in goats so far [15-20] and all used breeds/populations not included in our CNV study, except one in which a few Saanen crosses were analysed [15]. In addition, the identified QTL have very large confidence intervals including most of the CNVRs we observed in several chromosomes (data not shown). Therefore a comparison among CNVRs we identified in goats, their gene content and QTL regions is not very informative. However, it is interesting to note that Bolormaa et al. [20] reported putative QTL for faecal worm egg and eosinophil counts on CHI23, in the region including the major histocompatibility complex (MHC), in which we reported two CNVRs, one affecting a MHC class I antigen gene (CNVR no. 108, gain of copies) and the other one including MHC class II alpha and beta chain genes (CNVR no. 107, loss of copies) (Additional file 8 and Additional file 9). In sheep, several reports have identified polymorphisms in the class I and class II regions of the MHC being associated with resistance to nematodes [90]. It will be interesting to evaluate if these CNVs we identified in goats are associated with resistance to nematode infection and other diseases.

## Conclusions

We provide a first comparative map of CNVRs in the goat genome using a cross-species aCGH experiment based on the cattle genome. Considering the limited number of analysed animals and breeds and the fact that the cross-species hybridization could have limited the detection power of this study, the reported goat CNVRs largely underestimate the true number of this kind of variation in the goat genome. Additional studies including other breeds should be carried out in order to better evaluate the extension and distribution of CNVs in the genome of this farm animal species. However, it appeared that possible evolutionary conserved mechanisms might be the causative factors of putative recurrent interspecies CNVs between cattle and goat. Using this cross-species design it seems possible to analyse several other genomes of the *Bovidae *family in order to obtain comparative CNV maps in other species close to the cattle and provide additional evidence on the co-occurrence of CNVs in particular chromosome regions. Several goat CNVs affect genes with important biological functions. Further studies are needed to evaluate the functional relevance of these CNVs and their effects on behavior, production, and disease resistance traits in goats.

## Methods

### Array CGH

We analysed CNVs in the goat genome by means of a cross-species aCGH experiment using the Roche NimbleGen platform (Roche NimbleGen Inc., Madison, WI; http://www.nimblegen.com) based on custom tiling arrays designed on the bovine (*Bos taurus*) genome, Btau_4.0 version [74], including a fraction of BTA13 of the University of Maryland (UMD) *Bos taurus *v. 2.0 assembly (ftp://ftp.cbcb.umd.edu/pub/data/Bos_taurus/Bos_taurus_UMD_2.0/[75]). Arrays contained ~385,000 probes on a single slide to provide an evenly distributed coverage with an average interval of ~6 kb for the Btau_4.0 genome. The BTA13 of the UMD v. 2.0 assembly was included from nucleotide position 48 M bp to nucleotide position 78 M bp (4,673 oligonucleotides and average spacing of ~6 kb). This chromosome region was analysed as internal control because it contains the *ASIP *gene, not assembled in the BTA13 of the Btau_4.0 version. We previously showed that this goat gene is included in a CNVRs in different goat breeds [22].

Goat genomic DNA was extracted from blood of 2 Camosciata delle Alpi, 3 Girgentana, 3 Saanen, 1 black and 1 brown Murciano-Granadina goats using the Wizard^® ^Genomic DNA Purification kit (Promega Corporation, Madison, WI). All analysed animals were females. Reference DNA sample of one Camosciata delle Alpi goat (C1) was labeled with Cy5 and co-hybridised with the other test DNA samples labeled with Cy3 on 9 different arrays. A self hybridisation (reference labeled by both Cy5 and Cy3) was carried out in another array. Hybridization and array scanning were performed by Roche NimbleGen as previously described [39]. Data normalization was conducted using the normalize.qsline method from the Bioconductor package in R [39]. Then data were analysed for each hybridization using normalized log_2 _ratios using the CGHweb server (http://compbio.med.harvard.edu/CGHweb/[77]) that includes multiple algorithms. We used the self-self hybridisation and the BTA13 control region to define a suitable threshold to apply to the CGHweb calls in order to minimize false positives. Specifically we retained predicted CNVs if it had at least five consecutives datapoints supporting it (considering an average of probe values inside a smoothing window of five), thus limiting the minimum CNV size to about 30 kb, even if this resolution can vary in different regions depending on the relative distance of the probes that can be different from the averaged ~6 kb. Pointwise averaging of all computed profiles and maps of gains/losses for smoothed/segmented obtained from several algorithms (Lowess, Wavelet, Quantreg, ruavg, CBS, CGHseg, BioHMM, cghFLasso, GLAD, and FASeg) and summary data were generated. Pointwise averaging was shown to have good performances in calling alteration of copy number [91] and was chosen to compensate possible signal differences among probes in the cattle-goat heterologous experiment. Summary data were considered to call gain/loss in a chromosome region and to compile a high confidence set of CNVs. Then CNVs were called considering a conservative approach joining regions of at least 4-5 contiguous probes with CNV signal separated by up to three probes without CNV signal in the same individual (Additional file 1). This adjustment was applied in order to overcome possible signal losses or hybridisation problems in the cross-species aCGH experiment. CNVRs were reported aggregating overlapping or partially overlapping CNVs in different animals as previously reported [28,51] and applying the same criteria for CNVs within individuals (Additional file 2). The false discovery rate (FDR) was estimated based on the observation of 2 false positives in the self-self hybridisation. A rough estimate of the FDR is the expected number of false positives per array (n. 2) times the number of total arrays divided by the total number of CNVs (n. 161), resulting in an estimated FDR of 11%. This calculation should be considered only an approximation because it does not consider the potential for varying false positive rates across arrays. Based on these criteria the averaged log_2 _ratio threshold to call gains and losses [77] was empirically established at 0.175 considering the results obtained for the *ASIP *gene region. Four goats out of five with independent validated CNV [22] in this gene reported an averaged log_2 _ratio > 0.175, therefore this value was used as threshold even if in another goat the averaged log_2 _ratio for the *ASIP *region was 0.156. However, even if this latter value did not change the self-self FDR results, we used as threshold the value of 0.175 because the self-self hybridization could not fully reflect the variance of our 9 test experiments and we preferred a low false-positive rate even at the expense of having more false negatives in our dataset.

### Validation of CNVs

Validation of CNVs was performed by semiquantitative fluorescent multiplex PCR (SQF-PCR) as previously reported [22,92] using genomic DNA of the same goats analysed in the aCGH experiment and genomic DNA of additional goats (additional 8 Saanaen, 12 Girgentana, and 14 Murciano-Granadina) extracted as reported above. Briefly, two internal control regions known to have no CNV (*DGAT1 *and *MC1R *gene fragments) and CNVRs of interest were co-amplified in multiplex PCR under quantitative PCR conditions (with forward primers labelled in 5' with 6FAM) and the products were separated by capillary electrophoresis using an ABI3100 Avant sequencer (Applied Biosystems, Foster City, CA, USA) [22]. Peak heights of regions of interest were normalized against those of the internal controls. Primer pairs for control gene fragments and analysed CNVRs are reported in Additional file 6. SQF-PCR was performed in a total volume of 10 μL using 1-6 pmol of each primer and the conditions reported in Additional file 6. PCR profile was as follows: 5 min at 95°C; 20-22 amplification cycles of 30 sec at 95°C, 30 sec at 59°C, 30 sec at 72°C; 5 min at 72°C. Capillary electrophoresis was performed using 1 μL of reaction product, diluted in 10 μL of Hi-Di formamide (Applied Biosystems), and added with 0.1 μL of Rox labelled DNA ladder (500HD Rox, Applied Biosystems). Peak heights were obtained using GeneScan software v. 3.7 (Applied Biosystems). DNA dosages were calculated by comparing the normalized peak height ratios of goats of interest with the average normalized ratios of the reference Camosciata delle Alpi goat as follows: the peak height of a fragment of interest was divided by the peak height of the internal control; the averaged value obtained from at least two analyses for each sample/target region was divided by the same averaged value obtained for the control goat DNA. We adopted the theoretical values of 1.5, 2.0, 2.5, and so on for a gain of multiple of one, two, three or other copies, respectively, compared to the copy content (unknown) of the reference DNA sample. Similarly, a loss of one set of copies (or one copy in case of a simple duplication) would theoretically result in a value of 0.5. These values should be considered only approximation of the copy number content as the objective was to validate the results obtained with aCGH and not to precisely estimate the number of copies of the analysed DNA fragments.

### Bioinformatic and computational analyses

*Capra hircus *genomic sequences longer than 1 kb and including complete coding sequences were retrieved from EMBL database (Sept. 2010). Sequences were clustered with BLASTclust http://blast.ncbi.nlm.nih.gov/ on the basis of their identity (> 98%) resulting in 30 sequences covering on the whole 196,665 bp. These sequences were aligned with homologous cattle transcript regions identified using BLASTN on the basis of the best hits. The global sequence alignment without end-gap penalty was performed with LALIGN program http://www.ch.embnet.org/software/LALIGN_form.html. Exonic regions in goat sequences were defined according to the cattle annotation of the Btau_4.0 genome version http://www.ensembl.org/Bos_taurus/Info/Index. The goat CNVRs were mapped on the Btau_4.0 version of the bovine genome. To determine whether goat and cattle CNVRs occur in orthologous regions more often than expected by chance we considered the data reported for cattle in four different experiments [49-52]. The data reported in these four studies were considered separately due to differences in the methods and populations used for CNV detection. A merged list of the CNVRs reported in these investigations was also compiled (Additional file 4). In one of these cattle studies [48], CNVs were reported with reference to the Btau_3.0 version, therefore the LiftOver tool at the UCSC Genome Bioinformatics Site http://genome.ucsc.edu/cgi-bin/hgLiftOver was used to map CNVs coordinates on the Btau_4.0 version. In this case, only 45 out of the reported 79 CNVs were successfully re-mapped. Within each experiment, overlapping CNVs were fused to define CNVRs. These procedures ended up with 37, 368, 177, and 266 CNVRs for Matukumalli et al. [49], Bae et al. [50], Liu et al. [51], and Fadista et al. [52] experiments, respectively, for a total of 764 combined CNVRs (Additional file 4). The number of overlapping segments between each pair of CNVR sets was computed and the overlap significance was evaluated with a permutation test [37]. For each experiment, 10,000 artificial random rearrangements of the CNVRs were generated and mapped on the Btau_4.0 bovine genome. The CNVR length distribution was preserved in each random rearrangement. In order to evaluate the significance of the overlap between two CNVR sets, we computed the distribution of the number of overlapping segments between one of the CNVR sets and the 10,000 random rearrangements of the other one. The reported P-value is the fraction of random CNVR rearrangements that obtain at least the same number of overlapping segments as the real one.

Goat CNVRs superimposing with cattle transcripts annotated in the Btau_4.0 version were determined on the basis of the genome coordinates, without imposing a minimum overlap threshold. Gene ontology terms associated with bovine transcripts were downloaded with the Ensembl BioMart retrieval system http://www.ensembl.org/biomart/index.html and the complete annotation was obtained by reconstructing the complete list of ancestors of each term in the directed acyclic graph described by the OBO file downloaded from the Gene Ontology web site on May 2010 http://www.geneontology.org/. The GOTermFinder tool was adopted for this task http://search.cpan.org/dist/GO-TermFinder/. We computed the occurrence of each term in the set of transcripts overlapping with goat CNVRs and we compared it with the occurrence of the same term in the whole bovine genome (Btau_4.0 version). The Fisher exact test was adopted to assess the significance of the overrepresentation of the terms in the set of transcripts overlapping with the goat CNVRs. The multiple-hypothesis correction [93] was adopted for discriminating the significant terms at different False Discovery Rates (FDR): 0.001, 0.01, 0.05, and 0.1.

To supplement the functional annotation, PANTHER Molecular Function terms were assigned to all bovine transcripts using the Hidden Markov Model scoring tools of the PANTHER (Protein ANalysis THrough Evolutionary Relationships) Classification System version 6.1 http://panther6.ai.sri.com/tools/hmmScoreForm.jsp. Similarly to the GO annotation, the distribution of the PANTHER terms in the set of transcripts overlapping with goat CNVRs was compared with the occurrence in the whole genome and the significance of the overrepresentation was evaluated with the Fisher exact test adopting the multiple-hypothesis correction.

aCGH data have been submitted to the gene expression omnibus http://www.ncbi.nlm.nih.gov/geo/ under the accession number GSE24436.

## Authors' contributions

LF conceived and designed the study, analysed data, contributed to the sampling, coordinated and organized the laboratory work, drafted the manuscript and partially funded the study. PLM conducted bioinformatic and computational analyses. FB carried out the laboratory work and prepared data for analyses. VRi and SD contributed to the sampling and data. MC collaborated in the laboratory work. RC and VRu supervised the work and were involved in the design of the study. BP provided samples, supervised, took part in designing the work and partially funded the study. All authors reviewed the manuscript and accepted the final version.

## Supplementary Material

Additional file 1 **List of CNVs identified in the analysed goats**. The Excel file reports the chromosome, the nucleotides position of the CNV start and end (referred to the Btau_4.0 genome assembly), the size of the CNV in bp, the number of valid probes in the CNV (additional probes are included in CNV considering the position between two contiguous regions without 0.175 log_2 _value; see Methods for the definition of CNV), log_2 _mean of the probes in the CNV (see Methods), the type of CNV (gain/loss), the goat sample (C = Camosciata delle Alpi; G = Girgentana; MG = Murciano-Granadina; S = Saanaen; numbers after the breed symbols indicate the different animals used in the aCGH experiment), and the goat breed. Click here for file

Additional file 2 **List of CNVRs obtained by merging overlapping CNVs across animals**. The Excel file reports the progressive CNVR number, the chromosome, the nucleotides position of the CNVR start and end (referred to the Btau_4.0 genome assembly), the size of the CNVR in bp, the number of valid probes in the CNVR (additional probes are included in CNVR considering the position between two contiguous regions without 0.175 log_2 _value; see Methods for the definition of CNVR), the type of CNVR (gain/loss), the frequency of CNVR in the analysed goat panel, the goat breed (C = Camosciata delle Alpi; G = Girgentana; MG = Murciano-Granadina; S = Saanaen), and the goat subject (numbers after the breed symbols indicate the different animals used in the aCGH experiment). Click here for file

Additional file 3 **Extension of CNVRs in the different chromosomes**. Proportion of the CNVRs identified in goat compared to the dimension of the bovine chromosomes. Click here for file

Additional file 4 **List of cattle CNVRs reported in four other experiments **[49-52]**and overlapping with goat CNVRs**. CNVRs identified in cattle have been merged from the four reported experiments [49-52]. Progressive CNVR number has been assigned using the complete list. CNVRs are indicated with nucleotide positions (begin and end) on the Btau_4.0 version. Information reported for the four different experiments includes the progressive number and in parenthesis the chromosome number and the nucleotide positions (start and end). The goat CNVRs are reported. Click here for file

Additional file 5 **Tables reporting the P values for the overlapping between goat and cattle CNVRs and among the four CNVR datasets available in cattle**. Table S1 reports the results obtained comparing the goat CNVRs with the cattle CNVRs. Table S2 reports the results obtained comparing the different cattle datasets. Click here for file

Additional file 6 **Primers and PCR conditions used to validate goat CNVRs**. The table includes the goat CNVRs number, the corresponding bovine chromosome, gene symbol, amplified gene fragment data (including Ensembl number), sequence of the PCR primers, length of the amnplified fragment and PCR conditions. Click here for file

Additional file 7 **Semiquantitative fluorescent multiplex-PCR (SQF-PCR) results obtained for different goats**. The averaged SQF-PCR ratio normalized against the reference Camosciata delle Alpi goat is reported for the goats of the aCGH panel and for additional goats (additional panel) for the validated CNVs. Click here for file

Additional file 8 **Gene Ontology (GO) annotation of genes included in goat CNVRs**. Ensembl cattle transcripts located in goat CNVRs have been annotated using GO for Biological process, Cellular component, and Molecular function. Click here for file

Additional file 9 **PANTHER annotation of genes included in goat CNVRs**. Ensembl cattle transcripts located in goat CNVRs have been annotated using PANTHER. Click here for file

Additional file 10 **Gene ontology (GO) categories significantly overrepresented in goat CNVRs at different False Discovery Rate (FDR) levels**. GO categories were Molecular function, Biological process, and Cellular component. Click here for file

Additional file 11 **PANTHER categories significantly overrepresented in goat CNVRs**. PANTHER annotation has been obtained for the whole cattle genome. Click here for file

Additional file 12 **List of goat CNVRs with human orthologous genes**. EntrezGene ID and gene name are reported for human orthologous genes. The file includes genes already mapped in goat and genes for which mutations in human cause or are associated with human genetic diseases (data have been retrieved from OMIM database, May 2010). Click here for file
