# Supplementary material for: An initial comparative map of copy number variations in the goat (Capra hircus) genome
Source: BMC Genomics. 2010 Nov 17;11:639. doi: 10.1186/1471-2164-11-639 (PMC3011854; doi:10.1186/1471-2164-11-639)
Supplement: Additional file 5 — Tables reporting the P values for the overlapping between goat and cattle CNVRs and among the four CNVR datasets available in cattle. Table S1 reports the results obtained comparing the goat CNVRs with the cattle CNVRs. Table S2 reports the results obtained comparing the different cattle datasets. [file 1471-2164-11-639-S5.DOC]

**Table S1. P values derived from the permutation tests estimating the significance of the number of overlaps between goat CNVRs and different cattle CNVRs sets.**

|  | Bae et al. [50] | Matukumalli et al. [49] | Liu et al. [51] | Fadista et al. [52] | Merge^2^ |
| --- | --- | --- | --- | --- | --- |
| Goat | 0.8 (2)^1^ | 0.3 (2) | **< 10^-4^ (17)** | **< 10^-4^ (11)** | **< 10^-4^ (25)** |

^1^The number between parenthesis indicates the number of actual overlaps.

^2^Analysis including the merged CNVRs from the four cattle experiments (see Additional file 4).

**Table S2. P values** derived from the permutation tests estimating the significance of the number of overlaps between different cattle CNVRs sets.

| **Randomized trials** | | | | | |
| --- | --- | --- | --- | --- | --- |
| **Real CNVRs** |  | Bae et al. [50] | Matukumalli et al. [49] | Liu et al. [51] | Fadista et al. [52] |
|  | Bae et al. [50] | - | **2x10^-3^ (10)** | 0.8 (5) | 0.19 (15) |
|  | Matukumalli et al. [49] | **6x10^-3^ (10)**^1^ | - | **4x10^-3^ (7)** | 0.02 (4) |
|  | Liu et al. [51] | 0.8 (5) | **3x10^-3^ (7)** | - | **< 10^-4^ (50)** |
|  | Fadista et al. [52] | 0.14 (15) | 0.08 (4) | **< 10^-4^  (50)** | - |

^1^The number between parenthesis indicates the number of actual overlaps.
