# Supplementary material for: An initial comparative map of copy number variations in the goat (Capra hircus) genome
Source: BMC Genomics. 2010 Nov 17;11:639. doi: 10.1186/1471-2164-11-639 (PMC3011854; doi:10.1186/1471-2164-11-639)
Supplement: Additional file 6 — Primers and PCR conditions used to validate goat CNVRs. The table includes the goat CNVRs number, the corresponding bovine chromosome, gene symbol, amplified gene fragment data (including Ensembl number), sequence of the PCR primers, length of the amnplified fragment and PCR conditions. [file 1471-2164-11-639-S6.DOC]

## Additional file 6

## Primers and PCR conditions used to validate goat CNVRs

| CNVR no. | Chromosome | Gene | Amplified gene region^1^ (sequence) | Forward and *reverse* primers (5’-3’)^2^ | Fragment length (bp) | PCR conditions^3^ |
| --- | --- | --- | --- | --- | --- | --- |
| 25 | 4 | *GIMAP1* | Part of exon 1 (Btau_4.0: ENSBTAG00000001198, *Bos taurus*) | tttggacaatgcggatgtaa  *ttggttgttctgcaccatgt* | 157 | 59/2.0/24/1.0 |
| 61/62 | 10 | - | Part of exon 2 (Btau_4.0: ENSBTAG00000027170, *Bos taurus*) | gctctcctgaccctgaactg  *tacacagccgagtctgatgc* | 218 | 61/2.0/20/0.3 |
| 76 | 13 | *ASIP* | Part of intron 2, exon 3, and part of intron 3 (GenBank: AM746057, *Capra hircus*) | aaccctggggcttcctaga  *caggacggttttggtagctt* | 299 | 59/2.0/20/1.0 |
| 76 | 13 | *AHCY* | Part of the 3’-untranslated region (GenBank: EU185100, *Ovis aries*) | gcctggatggtaaagtgcat  *tctcctccccaagagcaaat* | 294 | 59/2.0/22/3.0 |
| 90 | 17 | *NR3C2* | Part of exon 5 (Btau_4.0: ENSBTAG00000027182, *Bos taurus*) | ggtgagcgacttgttggaat  *acggggaaacttaaggcaac* | 195 | 59/2.0/22/1.0 |
| Control | 14 | *DGAT1* | Part of intron 15, exon 16, intron 16 and part of exon 17 (GenBank: DQ380250, *Capra hircus*) | ccagtacctggtgagcatcc  *atgagtgacagccacacagc* | 215 | -/-/-/1.0 or 6.0 |
| Control | 18 | *MC1R* | Part of exon 1 (GenBank: FM212940, *Capra hircus*) | ctcgttggcctcttcatagc  *gaagttcttgaagatgcagcc* | 267 | -/-/-/1.0 or 6.0 |

^1^ Amplified fragments have been sequenced in order to confirm the amplification of the targeted regions (data not shown).

^2^ Primer forward was labelled at 5’ with 6FAM.

^3^ Annealing temperature (°C)/[MgCl_2_] (mM)/number of PCR cycles/primer concentration (pmol/µL); for the control PCR primers, annealing temperature, [MgCl_2_] and number of PCR cycles were the same as the CNVR tested primer pairs in the multiplex PCR analyses.
