# Supplementary material for: An initial comparative map of copy number variations in the goat (Capra hircus) genome
Source: BMC Genomics. 2010 Nov 17;11:639. doi: 10.1186/1471-2164-11-639 (PMC3011854; doi:10.1186/1471-2164-11-639)
Supplement: Additional file 10 — Gene ontology (GO) categories significantly overrepresented in goat CNVRs at different False Discovery Rate (FDR) levels. GO categories were Molecular function, Biological process, and Cellular component. [file 1471-2164-11-639-S10.DOC]

**Additional file 10**

Gene ontology (GO) categories significantly overrepresented in goat CNVRs at different False Discovery Rate (FDR) levels.

**Molecular Function**

| **GO term** | **GO name** | **FDR** | **No. in goat CNVRs** | **Expected number** |
| --- | --- | --- | --- | --- |
| GO:0005525 | GTP binding | <0.001 | 24 | 3.7 |
| GO:0032561 | guanyl ribonucleotide binding | <0.001 | 24 | 3.8 |
| GO:0003924 | GTPase activity | <0.001 | 16 | 2.1 |
| GO:0019001 | guanyl nucleotide binding | <0.001 | 24 | 3.9 |
| GO:0017111 | nucleoside-triphosphatase activity | <0.001 | 25 | 8.4 |
| GO:0016462 | pyrophosphatase activity | <0.001 | 25 | 8.6 |
| GO:0016818 | hydrolase activity, acting on acid anhydrides, in phosphorus-containing anhydrides | <0.001 | 25 | 8.6 |
| GO:0016817 | hydrolase activity, acting on acid anhydrides | <0.001 | 25 | 8.8 |
| GO:0015171 | amino acid transmembrane transporter activity | <0.001 | 6 | 0.50 |
| GO:0004869 | cysteine-type endopeptidase inhibitor activity | <0.001 | 5 | 0.37 |
| GO:0017076 | purine nucleotide binding | <0.001 | 37 | 19 |
| GO:0046943 | carboxylic acid transmembrane transporter activity | <0.001 | 6 | 0.73 |
| GO:0005342 | organic acid transmembrane transporter activity | <0.001 | 6 | 0.74 |
| GO:0042974 | retinoic acid receptor binding | <0.001 | 3 | 0.094 |
| GO:0046965 | retinoid X receptor binding | <0.001 | 3 | 0.094 |
| GO:0022804 | active transmembrane transporter activity | <0.001 | 12 | 3.3 |
| GO:0032553 | ribonucleotide binding | <0.001 | 37 | 18 |
| GO:0032555 | purine ribonucleotide binding | <0.001 | 37 | 18 |
| GO:0005275 | amine transmembrane transporter activity | <0.001 | 6 | 0.55 |
| GO:0003707 | steroid hormone receptor activity | <0.01 | 5 | 0.53 |
| GO:0004879 | ligand-dependent nuclear receptor activity | <0.01 | 5 | 0.55 |
| GO:0004089 | carbonate dehydratase activity | <0.01 | 3 | 0.13 |
| GO:0050542 | icosanoid binding | <0.01 | 2 | 0.034 |
| GO:0050543 | icosatetraenoic acid binding | <0.01 | 2 | 0.034 |
| GO:0050544 | arachidonic acid binding | <0.01 | 2 | 0.034 |
| GO:0000166 | nucleotide binding | <0.01 | 37 | 22 |
| GO:0019209 | kinase activator activity | <0.05 | 3 | 0.18 |
| GO:0031177 | phosphopantetheine binding | <0.05 | 3 | 0.23 |
| GO:0030296 | protein tyrosine kinase activator activity | <0.05 | 2 | 0.068 |
| GO:0033613 | transcription activator binding | <0.05 | 2 | 0.068 |
| GO:0003700 | transcription factor activity | <0.05 | 17 | 7.8 |
| GO:0004519 | endonuclease activity | <0.05 | 5 | 0.96 |
| GO:0031406 | carboxylic acid binding | <0.05 | 5 | 1.1 |
| GO:0016836 | hydro-lyase activity | <0.1 | 3 | 0.34 |
| GO:0016563 | transcription activator activity | <0.1 | 7 | 2.1 |
| GO:0004872 | receptor activity | <0.1 | 18 | 9.3 |
| GO:0035257 | nuclear hormone receptor binding | <0.1 | 3 | 0.41 |
| GO:0008144 | drug binding | <0.1 | 2 | 0.14 |
| GO:0030295 | protein kinase activator activity | <0.1 | 2 | 0.15 |
| GO:0016493 | C-C chemokine receptor activity | <0.1 | 2 | 0.15 |
| GO:0019957 | C-C chemokine binding | <0.1 | 2 | 0.15 |
| GO:0016835 | carbon-oxygen lyase activity | <0.1 | 3 | 0.45 |
| GO:0051427 | hormone receptor binding | <0.1 | 3 | 0.45 |

146 transcripts in goat CNVRs out of 249 are endowed with a GO annotation for the Molecular function.

17,077 transcripts in the cattle genome (Btau_4.0 version) out of 26,978 are endowed with a GO annotation for the Molecular function.

Biological Process

| **GO term** | **GO name** | **FDR** | **No. in goat CNVRs** | **Expected number** |
| --- | --- | --- | --- | --- |
| GO:0050896 | response to stimulus | <0.001 | 41 | 15 |
| GO:0006952 | defense response | <0.001 | 16 | 2.6 |
| GO:0006950 | response to stress | <0.001 | 25 | 8.0 |
| GO:0051704 | multi-organism process | <0.001 | 13 | 2.2 |
| GO:0046942 | carboxylic acid transport | <0.001 | 8 | 0.76 |
| GO:0015849 | organic acid transport | <0.001 | 8 | 0.77 |
| GO:0051707 | response to other organism | <0.001 | 10 | 1.5 |
| GO:0009617 | response to bacterium | <0.001 | 9 | 1.1 |
| GO:0042742 | defense response to bacterium | <0.001 | 7 | 0.68 |
| GO:0019882 | antigen processing and presentation | <0.01 | 7 | 0.79 |
| GO:0006865 | amino acid transport | <0.01 | 6 | 0.54 |
| GO:0009607 | response to biotic stimulus | <0.01 | 10 | 1.9 |
| GO:0015837 | amine transport | <0.01 | 6 | 0.65 |
| GO:0045730 | respiratory burst | <0.01 | 3 | 0.061 |
| GO:0002376 | immune system process | <0.01 | 16 | 5.1 |
| GO:0032799 | low-density lipoprotein receptor metabolic process | <0.01 | 2 | 0.015 |
| GO:0045713 | low-density lipoprotein receptor biosynthetic process | <0.01 | 2 | 0.015 |
| GO:0055098 | response to low-density lipoprotein stimulus | <0.01 | 2 | 0.023 |
| GO:0001818 | negative regulation of cytokine production | <0.01 | 4 | 0.29 |
| GO:0032594 | protein transport within lipid bilayer | <0.05 | 2 | 0.030 |
| GO:0032599 | protein transport out of membrane raft | <0.05 | 2 | 0.030 |
| GO:0032600 | chemokine receptor transport out of membrane raft | <0.05 | 2 | 0.030 |
| GO:0032800 | receptor biosynthetic process | <0.05 | 2 | 0.030 |
| GO:0032910 | regulation of transforming growth factor-beta3 production | <0.05 | 2 | 0.030 |
| GO:0032913 | negative regulation of transforming growth factor-beta3 production | <0.05 | 2 | 0.030 |
| GO:0033606 | chemokine receptor transport within lipid bilayer | <0.05 | 2 | 0.030 |
| GO:0010887 | negative regulation of cholesterol storage | <0.05 | 2 | 0.038 |
| GO:0010891 | negative regulation of sequestering of triglyceride | <0.05 | 2 | 0.038 |
| GO:0030224 | monocyte differentiation | <0.05 | 2 | 0.038 |
| GO:0055094 | response to lipoprotein stimulus | <0.05 | 2 | 0.038 |
| GO:0071635 | negative regulation of transforming growth factor-beta production | <0.05 | 2 | 0.038 |
| GO:0010551 | regulation of gene-specific transcription from RNA polymerase II promoter | <0.05 | 5 | 0.73 |
| GO:0009719 | response to endogenous stimulus | <0.05 | 6 | 1.1 |
| GO:0045600 | positive regulation of fat cell differentiation | <0.05 | 2 | 0.045 |
| GO:0010552 | positive regulation of gene-specific transcription from RNA polymerase II promoter | <0.05 | 4 | 0.45 |
| GO:0010871 | negative regulation of receptor biosynthetic process | <0.05 | 2 | 0.053 |
| GO:0033993 | response to lipid | <0.05 | 2 | 0.053 |
| GO:0050872 | white fat cell differentiation | <0.05 | 2 | 0.053 |
| GO:0002573 | myeloid leukocyte differentiation | <0.05 | 3 | 0.22 |
| GO:0010889 | regulation of sequestering of triglyceride | <0.05 | 2 | 0.061 |
| GO:0042953 | lipoprotein transport | <0.05 | 2 | 0.061 |
| GO:0010745 | negative regulation of macrophage derived foam cell differentiation | <0.05 | 2 | 0.068 |
| GO:0010885 | regulation of cholesterol storage | <0.05 | 2 | 0.068 |
| GO:0071634 | regulation of transforming growth factor-beta production | <0.05 | 2 | 0.068 |
| GO:0015909 | long-chain fatty acid transport | <0.1 | 2 | 0.076 |
| GO:0006955 | immune response | <0.1 | 9 | 3.0 |
| GO:0009725 | response to hormone stimulus | <0.1 | 5 | 0.97 |
| GO:0032583 | regulation of gene-specific transcription | <0.1 | 5 | 0.98 |
| GO:0010869 | regulation of receptor biosynthetic process | <0.1 | 2 | 0.084 |
| GO:0043193 | positive regulation of gene-specific transcription | <0.1 | 4 | 0.62 |
| GO:0010553 | negative regulation of gene-specific transcription from RNA polymerase II promoter | <0.1 | 3 | 0.32 |
| GO:0051179 | localization | <0.1 | 34 | 22 |
| GO:0001959 | regulation of cytokine-mediated signaling pathway | <0.1 | 2 | 0.099 |
| GO:0010888 | negative regulation of lipid storage | <0.1 | 2 | 0.099 |
| GO:0006810 | transport | <0.1 | 31 | 19 |
| GO:0051234 | establishment of localization | <0.1 | 31 | 20 |
| GO:0031294 | lymphocyte costimulation | <0.1 | 2 | 0.11 |
| GO:0031295 | T cell costimulation | <0.1 | 2 | 0.11 |
| GO:0010743 | regulation of macrophage derived foam cell differentiation | <0.1 | 2 | 0.11 |
| GO:0042104 | positive regulation of activated T cell proliferation | <0.1 | 2 | 0.11 |
| GO:0045598 | regulation of fat cell differentiation | <0.1 | 2 | 0.11 |
| GO:0007165 | signal transduction | <0.1 | 22 | 13 |
| GO:0002504 | antigen processing and presentation of peptide or polysaccharide antigen via MHC class II | <0.1 | 2 | 0.12 |
| GO:0015908 | fatty acid transport | <0.1 | 2 | 0.12 |

124 transcripts in goat CNVRs out of 249 are endowed with a GO annotation for the Biological Process.

16,306 transcripts in the cattle genome (Btau_4.0 version) out of 26,978 are endowed with a GO annotation for the Biological Process.

Cellular Component

| **GO term** | **GO name** | **FDR** | **No. in goat CNVRs** | **Expected number** |
| --- | --- | --- | --- | --- |
| GO:0042613 | MHC class II protein complex | <0.001 | 5 | 0.14 |
| GO:0042611 | MHC protein complex | <0.001 | 7 | 0.62 |
| GO:0005886 | plasma membrane | <0.05 | 26 | 13 |
| GO:0044459 | plasma membrane part | <0.05 | 18 | 7.5 |
| GO:0005576 | extracellular region | <0.1 | 19 | 9.6 |

122 transcripts in CNVRs out of 249 are endowed with a GO annotation for the Cellular Component.

16,123 transcripts in the cattle genome (Btau_4.0 version) out of 26,978 are endowed with a GO annotation for the Cellular Component.
