# Supplementary material for: An initial comparative map of copy number variations in the goat (Capra hircus) genome
Source: BMC Genomics. 2010 Nov 17;11:639. doi: 10.1186/1471-2164-11-639 (PMC3011854; doi:10.1186/1471-2164-11-639)
Supplement: Additional file 11 — PANTHER categories significantly overrepresented in goat CNVRs. PANTHER annotation has been obtained for the whole cattle genome. [file 1471-2164-11-639-S11.DOC]

**Additional file 11**

**PANTHER categories significantly overrepresented in goat CNVRs.**

| ***PANTHER Code*** | ***PANTHER Name*** | **SignificanceLevel** | ***No. in goat CNVRs*** | ***Expected no.*** |
| --- | --- | --- | --- | --- |
| PTHR23267 | IMMUNOGLOBULIN LIGHT CHAIN | <0.001 | 43 | 2.1 |
| PTHR23267_SF6 | gb def: TRA@ protein | <0.001 | 33 | 0.89 |
| PTHR19433 | T-CELL RECEPTOR ALPHA CHAIN V REGION-RELATED | <0.001 | 40 | 1.7 |
| PTHR23267_SF5 | gb def: Hypothetical protein | <0.001 | 27 | 0.65 |
| PTHR19433_SF2 | T-CELL RECEPTOR ALPHA CHAIN V REGION | <0.001 | 33 | 1.3 |
| PTHR19343 | FAMILY NOT NAMED | <0.001 | 29 | 0.96 |
| PTHR23267_SF4 | TRA PROTEIN | <0.001 | 30 | 1.29 |
| PTHR10751_SF2 | INTERFERON-INDUCED GUANYLATE-BINDING PROTEIN | <0.001 | 16 | 0.21 |
| PTHR10206 | CATHELICIDIN-RELATED | <0.001 | 13 | 0.15 |
| PTHR10751_SF3 | ATLASTIN | <0.001 | 16 | 0.30 |
| PTHR10751 | GUANYLATE-BINDING PROTEIN | <0.001 | 16 | 0.30 |
| PTHR23268_SF3 | T-CELL RECEPTOR ALPHA CHAIN V REGION | <0.001 | 17 | 0.75 |
| PTHR23267_SF3 | gb def: Hypothetical protein | <0.001 | 13 | 0.39 |
| PTHR19367 | T-CELL RECEPTOR ALPHA CHAIN V REGION | <0.001 | 14 | 0.65 |
| PTHR23268 | T-CELL RECEPTOR BETA CHAIN | <0.001 | 14 | 1.20 |
| PTHR19339 | FAMILY NOT NAMED | <0.001 | 8 | 0.33 |
| PTHR11785_SF53 | CATIONIC AMINO ACID TRANSPORTER | <0.001 | 6 | 0.18 |
| PTHR23097_SF41 | TUMOR NECROSIS FACTOR RECEPTOR SUPERFAMILY MEMBER 10 | <0.001 | 4 | 0.039 |
| PTHR23267_SF9 | IMMUNOGLOBULIN LAMBDA CHAIN VARIABLE REGION | <0.001 | 7 | 0.36 |
| PTHR10903_SF10 | GTPASE, IMAP FAMILY MEMBER 7 | <0.001 | 5 | 0.15 |
| PTHR10903_SF7 | GTPASE, IMAP FAMILY MEMBER 1 | <0.001 | 5 | 0.16 |
| PTHR23267_SF15 | IMMUNOGLOBULIN LAMBDA 2 LIGHT CHAIN VARIABLE REGION | <0.001 | 5 | 0.16 |
| PTHR10903 | AIG1 | <0.001 | 5 | 0.16 |
| PTHR11785 | AMINO ACID TRANSPORTER | <0.001 | 6 | 0.31 |
| PTHR10903_SF5 | GTPASE, IMAP FAMILY MEMBER 5 | <0.001 | 4 | 0.11 |
| PTHR11785_SF55 | gb def: Hypothetical transport protein ycjJ | <0.001 | 3 | 0.039 |
| PTHR13451 | CLASS II CROSSOVER JUNCTION ENDONUCLEASE MUS81 | <0.001 | 3 | 0.039 |
| PTHR19900_SF7 | TITIN | <0.001 | 4 | 0.13 |
| PTHR11774 | GERANYLGERANYL TRANSFERASE TYPE BETA SUBUNIT | <0.001 | 3 | 0.048 |
| PTHR11774_SF3 | TYPE I GERANYLGERANYLTRANSFERASE BETA SUBUNIT | <0.001 | 3 | 0.048 |
| PTHR11774_SF4 | GERANYLGERANYL TRANSFERASE TYPE I BETA SUBUNIT | <0.001 | 3 | 0.048 |
| PTHR11774_SF5 | GERANYLGERANYL TRANSFERASE TYPE II BETA SUBUNIT | <0.001 | 3 | 0.048 |
| PTHR11774_SF6 | PROTEIN FARNESYLTRANSFERASE BETA SUBUNIT (CAAX FARNESYLTRANSFERASE BETA SUBUNIT) (RAS PROTEINS PRENYLTRANSFERASE BETA) (FTASE-BETA) | <0.001 | 3 | 0.048 |
| PTHR11865_SF206 | PEROXISOME PROLIFERATOR ACTIVATED RECEPTOR ALPHA (PPAR-ALPHA) | <0.001 | 3 | 0.048 |
| PTHR11865_SF207 | PEROXISOME PROLIFERATOR ACTIVATED RECEPTOR DELTA (PPAR-DELTA) (PPAR- BETA) | <0.001 | 3 | 0.048 |
| PTHR11865_SF208 | gb def: Peroxisome proliferator activated receptor beta (PPAR-beta) | <0.001 | 3 | 0.048 |
| PTHR23097_SF20 | TUMOR NECROSIS FACTOR RECEPTOR RELATED | <0.001 | 3 | 0.048 |
| PTHR23097_SF22 | DEATH RECEPTOR | <0.001 | 3 | 0.048 |
| PTHR11774_SF6 | PROTEIN FARNESYLTRANSFERASE BETA SUBUNIT (CAAX FARNESYLTRANSFERASE BETA SUBUNIT) (RAS PROTEINS PRENYLTRANSFERASE BETA) (FTASE-BETA) | <0.001 | 3 | 0.048 |
| PTHR19944 | MHC CLASS II-RELATED | <0.001 | 5 | 0.31 |
| PTHR19944_SF26 | MHC CLASS II BETA CHAIN | <0.001 | 3 | 0.17 |
| PTHR23267_SF11 | IMMUNOGLOBULIN IOTA CHAIN | <0.001 | 4 | 0.41 |
| PTHR23267_SF33 | IMMUNOGLOBULIN KAPPA LIGHT CHAIN V REGION | <0.001 | 5 | 0.45 |
| PTHR23097 | TUMOR NECROSIS FACTOR RECEPTOR SUPERFAMILY MEMBER | <0.001 | 4 | 0.24 |
| PTHR18952_SF26 | CARBONIC ANHYDRASE VII (CARBONATE DEHYDRATASE VII) | <0.001 | 3 | 0.097 |
| PTHR18952_SF31 | CARBONIC ANHYDRASE XIII (CARBONATE DEHYDRATASE XIII) | <0.001 | 3 | 0.097 |
| PTHR19242_SF16 | gb def: Multidrug resistance protein family protein 6 | <0.001 | 4 | 0.26 |
| PTHR18952_SF28 | CARBONIC ANHYDRASE II (CARBONATE DEHYDRATASE II) | <0.001 | 3 | 0.11 |
| PTHR19944_SF12 | MHC CLASS II ALPHA CHAIN | <0.001 | 3 | 0.11 |
| PTHR11708_SF38 | RHO-RELATED BTB DOMAIN-CONTAINING PROTEIN | <0.001 | 3 | 0.058 |
| PTHR11865_SF209 | PEROXISOME PROLIFERATOR ACTIVATED RECEPTOR GAMMA (PPAR-GAMMA) | <0.001 | 3 | 0.058 |
| PTHR18952_SF29 | CARBONIC ANHYDRASE III (CARBONATE DEHYDRATASE III) | <0.001 | 3 | 0.058 |
| PTHR10555_SF5 | UNCHARACTERIZED | <0.01 | 3 | 0.068 |
| PTHR10555_SF6 | SORTING NEXIN 5 | <0.01 | 3 | 0.068 |
| PTHR10555_SF7 | SORTING NEXIN 6 | <0.01 | 3 | 0.068 |
| PTHR11785_SF54 | AMINO ACID TRANSPORTER | <0.01 | 3 | 0.068 |
| PTHR18952_SF30 | CARBONIC ANHYDRASE I (CARBONATE DEHYDRATASE I) | <0.01 | 3 | 0.077 |
| PTHR10903_SF9 | GTPASE, IMAP FAMILY MEMBER 4 | <0.01 | 3 | 0.077 |
| PTHR23097_SF37 | TUMOR NECROSIS FACTOR RECEPTOR SUPERFAMILY MEMBER 6B (DECOY RECEPTOR 3) | <0.01 | 3 | 0.077 |
| PTHR11785_SF4 | gb def: LOC284379 protein (Fragment) | <0.01 | 3 | 0.087 |
| PTHR19944_SF16 | BETA-2 MICROGLOBULIN | <0.01 | 3 | 0.087 |
| PTHR19242_SF8 | CYSTIC FIBROSIS TRANSMEMBRANE CONDUCTANCE REGULATOR | <0.01 | 4 | 0.29 |
| PTHR13633 | UNCHARACTERIZED | <0.05 | 2 | 0.019 |
| PTHR10845_SF24 | REGULATOR OF G PROTEIN SIGNALING | <0.05 | 2 | 0.029 |
| PTHR10845_SF25 | REGULATOR OF G PROTEIN SIGNALING | <0.05 | 2 | 0.029 |
| PTHR10845_SF27 | REGULATOR OF G PROTEIN SIGNALING 6, RGS6 | <0.05 | 2 | 0.029 |
| PTHR13336 | OVARIAN CARCINOMA IMMUNOREACTIVE ANTIGEN | <0.05 | 2 | 0.029 |
| PTHR21678 | GROWTH INHIBITION AND DIFFERENTIATION RELATED PROTEIN 88 | <0.05 | 2 | 0.029 |
| PTHR11708_SF290 | RHO-RELATED GTP-BINDING PROTEIN RHOQ (RAS-RELATED GTP-BINDING PROTEIN TC10) | <0.05 | 3 | 0.14 |
| PTHR11865 | NUCLEAR HORMONE RECEPTOR | <0.05 | 5 | 0.61 |
| PTHR10903_SF6 | GTPASE, IMAP FAMILY MEMBER 6 | <0.05 | 3 | 0.15 |
| PTHR10766_SF12 | TRANSMEMBRANE 9 SUPERFAMILY PROTEIN MEMBER 4 | <0.05 | 2 | 0.039 |
| PTHR10766_SF13 | TRANSMEMBRANE 9 SUPERFAMILY MEMBER 2 | <0.05 | 2 | 0.039 |
| PTHR11849_SF33 | ERG | <0.05 | 2 | 0.039 |
| PTHR11849_SF34 | FLI1 | <0.05 | 2 | 0.039 |
| PTHR11849_SF35 | ETS-RELATED | <0.05 | 2 | 0.039 |
| PTHR11865_SF173 | ORPHAN NUCLEAR RECEPTOR NR1D1 AND NR1D2 (V-ERBA RELATED PROTEIN EAR) | <0.05 | 3 | 0.17 |
| PTHR18952 | CARBONIC ANHYDRASE | <0.05 | 3 | 0.17 |
| PTHR19944_SF26 | MHC CLASS II BETA CHAIN | <0.05 | 3 | 0.17 |
| PTHR23267_SF11 | IMMUNOGLOBULIN IOTA CHAIN | <0.05 | 4 | 0.41 |
| PTHR23266 | IMMUNOGLOBULIN HEAVY CHAIN | <0.05 | 3 | 0.18 |
| PTHR10845_SF26 | REGULATOR OF G PROTEIN SIGNALING 7, RGS7 | <0.05 | 2 | 0.048 |
| PTHR11347_SF5 | CAMP-SPECIFIC 3,5-CYCLIC PHOSPHODIESTERASE 4 | <0.05 | 2 | 0.048 |
| PTHR11849_SF37 | ETS-RELATED | <0.05 | 2 | 0.048 |
| PTHR19242_SF97 | MULTIDRUG RESISTANCE-ASSOCIATED PROTEIN 4 (MRP/CMOAT-RELATED ABC TRANSPORTER) | <0.05 | 4 | 0.46 |
| PTHR11708_SF287 | RHO-RELATED GTP-BINDING PROTEIN RHOG | <0.05 | 3 | 0.22 |
| PTHR10766 | TRANSMEMBRANE 9 SUPERFAMILY PROTEIN MEMBER | <0.05 | 2 | 0.058 |
| PTHR11849_SF38 | PET-1 | <0.05 | 2 | 0.058 |
| PTHR19264_SF68 | G-PROTEIN COUPLED RECEPTOR 37 | <0.05 | 2 | 0.058 |
| PTHR16675_SF4 | MHC CLASS I-ALPHA | <0.05 | 3 | 0.23 |
| PTHR19944_SF27 | MHC CLASS II ALPHA CHAIN | <0.05 | 3 | 0.23 |
| PTHR11506 | LYSOSOME-ASSOCIATED MEMBRANE GLYCOPROTEIN | <0.1 | 2 | 0.068 |
| PTHR11506_SF5 | LYSOSOME-ASSOCIATED MEMBRANE GLYCOPROTEIN 1 | <0.1 | 2 | 0.068 |
| PTHR10555 | SORTING NEXIN | <0.1 | 3 | 0.26 |
| PTHR19900 | MYOSIN-BINDING PROTEIN-RELATED | <0.1 | 3 | 0.27 |
| PTHR11616_SF35 | SODIUM-DEPENDENT PROLINE TRANSPORTER | <0.1 | 2 | 0.077 |
| PTHR19944_SF13 | MHC CLASS II H2-M ALPHA CHAIN | <0.1 | 2 | 0.077 |
| PTHR23266_SF4 | IMMUNOGLOBULIN GAMMA HEAVY CHAIN CONSTANT REGION | <0.1 | 2 | 0.077 |
| PTHR19242_SF32 | METAL RESISTANCE PROTEIN YCF1 (YEAST CADMIUM FACTOR 1) | <0.1 | 3 | 0.28 |
| PTHR19242_SF28 | ABC TRANSPORTER (ABCC) | <0.1 | 3 | 0.29 |
| PTHR11616_SF34 | GLYCINE TRANSPORTER | <0.1 | 2 | 0.087 |
| PTHR19264_SF239 | BOMBESIN RECEPTOR | <0.1 | 2 | 0.087 |
| PTHR19264_SF69 | ENDOTHELIN RECEPTOR | <0.1 | 2 | 0.087 |
| PTHR23230_SF154 | KELCH-LIKE PROTEIN 12 | <0.1 | 2 | 0.087 |
| PTHR11708_SF285 | RAS-RELATED PROTEIN RAC | <0.1 | 3 | 0.30 |

232 transcripts in goat CNVRs out of 249 are endowed with at least a PANTHER Domain (E-value < 0.001). 870 different domain types are present in goat CNVRs.

23970 transcripts in the cattle genome (Btau_4.0 version) out of 26,978 are endowed with at least one PANTHER domain. 18842 domain types are present in the cattle genome (Btau_4.0 version).
